# Comparative transcriptome analysis uncovers the regulatory functions of long noncoding RNAs in fruit development and color changes of *Fragaria pentaphylla*

**DOI:** 10.1038/s41438-019-0128-4

**Published:** 2019-03-04

**Authors:** Lijun Bai, Qing Chen, Leiyu Jiang, Yuanxiu Lin, Yuntian Ye, Peng Liu, Xiaorong Wang, Haoru Tang

**Affiliations:** 1 College of Horticulture, Sichuan Agricultural University, Chengdu, 611130 China; 2 Chengdu Life Baseline Technology Co., LTD, Chengdu, China

**Keywords:** Plant genetics, Gene expression

## Abstract

To investigate the molecular mechanism underlying fruit development and color change, comparative transcriptome analysis was employed to generate transcriptome profiles of two typical wild varieties of *Fragaria pentaphylla* at three fruit developmental stages (green fruit stage, turning stage, and ripe fruit stage). We identified 25,699 long noncoding RNAs (lncRNAs) derived from 25,107 loci in the *F. pentaphylla* fruit transcriptome, which showed distinct stage- and genotype-specific expression patterns. Time course analysis detected a large number of differentially expressed protein-coding genes and lncRNAs associated with fruit development and ripening in both of the *F. pentaphylla* varieties. The target genes downregulated in the late stages were enriched in terms of photosynthesis and cell wall organization or biogenesis, suggesting that lncRNAs may act as negative regulators to suppress photosynthesis and cell wall organization or biogenesis during fruit development and ripening of *F. pentaphylla*. Pairwise comparisons of two varieties at three developmental stages identified 365 differentially expressed lncRNAs in total. Functional annotation of target genes suggested that lncRNAs in *F. pentaphylla* may play roles in fruit color formation by regulating the expression of structural genes or regulatory factors. Construction of the regulatory network further revealed that the low expression of *Fra a* and *CHS* may be the main cause of colorless fruit in *F. pentaphylla*.

## Ripening: What makes strawberries turn red?

Researchers have pinpointed key genes that make strawberries turn red when they ripen. The role of hormones in fruit ripening has been extensively studied, but questions remain about the underlying genetics. Haoru Tang at Sichuan Agricultural University in China and co-workers assembled detailed genetic profiles of two wild strawberry varieties, one with red and the other with white fruits, at three stages of ripening. They focused on long non-coding RNAs (lncRNAs), master switches of gene regulation. Identifying differences in lncRNA profiles between the varieties allowed them to trace the complex genetic networks controlling ripening and redness. They also identified two key genes that generate red pigment, which are switched off in the white fruits. These results illuminate the complex genetics underlying a key feature of one of the world’s most economically important fruits. Researchers have illuminated the genetic cues that control the ripening of strawberries by comparing the genes switched on two variants, that produce red or white fruits, respectively.

## Introduction

Strawberry (*Fragaria*) is considered one of the most economically important soft fruits in the world. *Fragaria* belongs to the family Rosaceae, which contains *Duchesnea* and *Potentilla* as close relatives^1,2^. This genus consists of approximately 24 species representing different ploidy levels, in which the wild members are mainly distributed in the Northern Hemisphere as well as western South America^3^. Fruit development and ripening of *Fragaria* are closely correlated with its economic value. Among other factors, plant hormones have crucial roles in regulating these processes. Numerous advances have been made in understanding the impact of auxin and gibberellins (GAs) on *Fragaria* fruit development and ripening^4–6^. It has been confirmed that the interaction between auxin and GA signaling pathways is essential for the promotion of fruit set and the early stage of fruit development^6^. The levels of indole-3-acetic acid (IAA) and bioactive GAs rose rapidly during the early phases of fruit development and then declined as ripening progressed, whereas abscisic acid (ABA) showed the opposite pattern in strawberry^7^. Auxin is already known to have an important impact on fruit growth and size as well as anthocyanin synthesis and, hence, the fruit ripening process^8,9^. Transcriptome profiling further revealed that auxin suppressed anthocyanin biosynthesis by downregulating genes involved in anthocyanin and flavonoid synthesis and transportation into the vacuole. In contrast, genes related to the auxin signaling pathway were upregulated, including Aux/IAA and auxin response factor (ARF)^10^. Recent results reveal that the upregulation of the *FveCYP707A4a* gene by GAs and auxin is a key cross-talk signal in the early fruit development stage to repress the elevation of ABA, which mainly acts in the ripening phase^11^. Furthermore, differentially expressed genes encoding proteins for phytohormones at various developmental stages provide evidence that other hormones, such as cytokinin, brassinosteroid (BR), and ethylene, are also involved in the fruit development and ripening of *Fragaria*^12^. Physiological and molecular evidence suggests that BR is associated with the early fruit development and ripening of *Fragaria*^13^. In addition, silencing of a 9-cis-epoxycarotenoid dioxygenase gene (*FaNCED1*), which functions in ABA biosynthesis, resulted in a significant decrease in ABA level and uncolored fruits^14^.

Fruit ripening is a complex biological process involving sugar accumulation, cell wall hydrolysis, flavor and aroma production as well as color changes^15^. Color change, which can be directly observed, is often used as a marker for ripening. Anthocyanins are the most prominent pigments in strawberry that not only contribute to fruit color modification but also have potential health benefits, which subsequently affect the aesthetic and commercial value of strawberry. It has been revealed that anthocyanins, flavonols, proanthocyanidins (PAs), and lignin are all derived from the phenylpropanoid pathway^16^. Moreover, miscellaneous enzyme families, transport proteins as well as transcription factors (TFs) construct complex and intricate biological networks that regulate anthocyanin synthesis. Some flavonoid biosynthetic pathway genes, including *cinnamate 4-hydroxylase* (*C4H*), *chalcone synthase* (*CHS*), *chalcone isomerase* (*CHI*), *flavanone 3-hydroxylase* (*F3H*), *dihydroflavonol-4-reductase* (*DFR*), and *anthocyanidin synthase* (*ANS*), showed lower expression abundances in yellow strawberry fruits than in red strawberry fruits, which were coordinated by genes encoding TFs, such as the MYB, bHLH, WD40, MADS-box, and WRKY families^17,18^. The members of the MYB family were previously identified and shown to play critical roles in the regulation of red pigment synthesis in *F.* *×* *ananassa* and *F. vesca*^19,20^. One SNP causing an amino acid change in the *FveMYB10* gene was identified in red and yellow *F. vesca* accessions and further confirmed to be responsible for the yellow color of fruits^20^. In addition, pathogenesis-related protein family 10 (PR-10) has received considerable attention for its potential to control anthocyanin formation^21,22^. *Fra a* allergen, one member of the PR10 family, was directly linked to flavonoid biosynthesis and had an essential biological function in strawberry fruit color formation^22^.

Non-coding RNAs with a length longer than 200 nucleotides are defined as long noncoding RNAs (lncRNAs). They are involved in chromatin modification, epigenetic regulation, genomic imprinting, transcriptional control as well as pre- and post-translational mRNA processing^23,24^. A large number of lncRNAs have been identified and characterized in plants. It has been revealed that lncRNAs play widespread roles in diverse biological processes in plants^25,26^. The function of COOLAIR and COLDAIR has been studied thoroughly in regulating flowering in *Arabidopsis*. The floral repressor *flowering locus C* (*FLC*) is repressed by COOLAIR and COLDAIR during the process of vernalization^27,28^. The intergenic lncRNA LDMAR (long-day-specific-male fertility-associated RNA) is required for normal pollen development in rice under long-day conditions and regulated by Psi-LDMAR through the RNA-dependent DNA methylation (RdDM) pathway^26,29^. Furthermore, a number of lncRNAs have been reported to participate in stress responses, such as *Mt4* in *Medicago truncatula*^30^, *IPS1* (induced by phosphate starvation 1) and *At4* in *A. thaliana*^31–33^, *TPSI1* (tomato phosphate starvation-induced gene 1) in *Solanum lycopersicum*^34^ and *OsPI1* (*Oryza sativa* phosphate starvation-induced gene 1) in *O. sativa*^35^, which were induced by phosphate starvation.

A growing number of reports have revealed that lncRNAs play vital roles in gene regulation and other biological processes in plants. However, the function of lncRNAs in strawberry fruit development and ripening processes, especially color change, is largely unexplored. Thus, we profiled the global gene expression patterns of two typical wild varieties of *F. pentaphylla* Lozinsk with different fruit colors at three developmental stages using high-throughput transcriptome sequencing. We identified lncRNAs from different samples using de novo assembly and genome-guided assembly. Differential expression of lncRNAs and comparative analysis between two varieties of *F. pentaphylla* were investigated to reveal the different regulation of key pathways. Furthermore, regulatory networks of the identified lncRNAs, mRNAs and miRNAs were constructed to elucidate the potential regulatory processes of lncRNAs. This study provides valuable molecular information for insight into the function of lncRNAs in *F. pentaphylla* during fruit development and ripening.

## Materials and methods

### *F. pentaphylla* fruit collection, RNA extraction, library construction, and sequencing

**Fig. 1:**
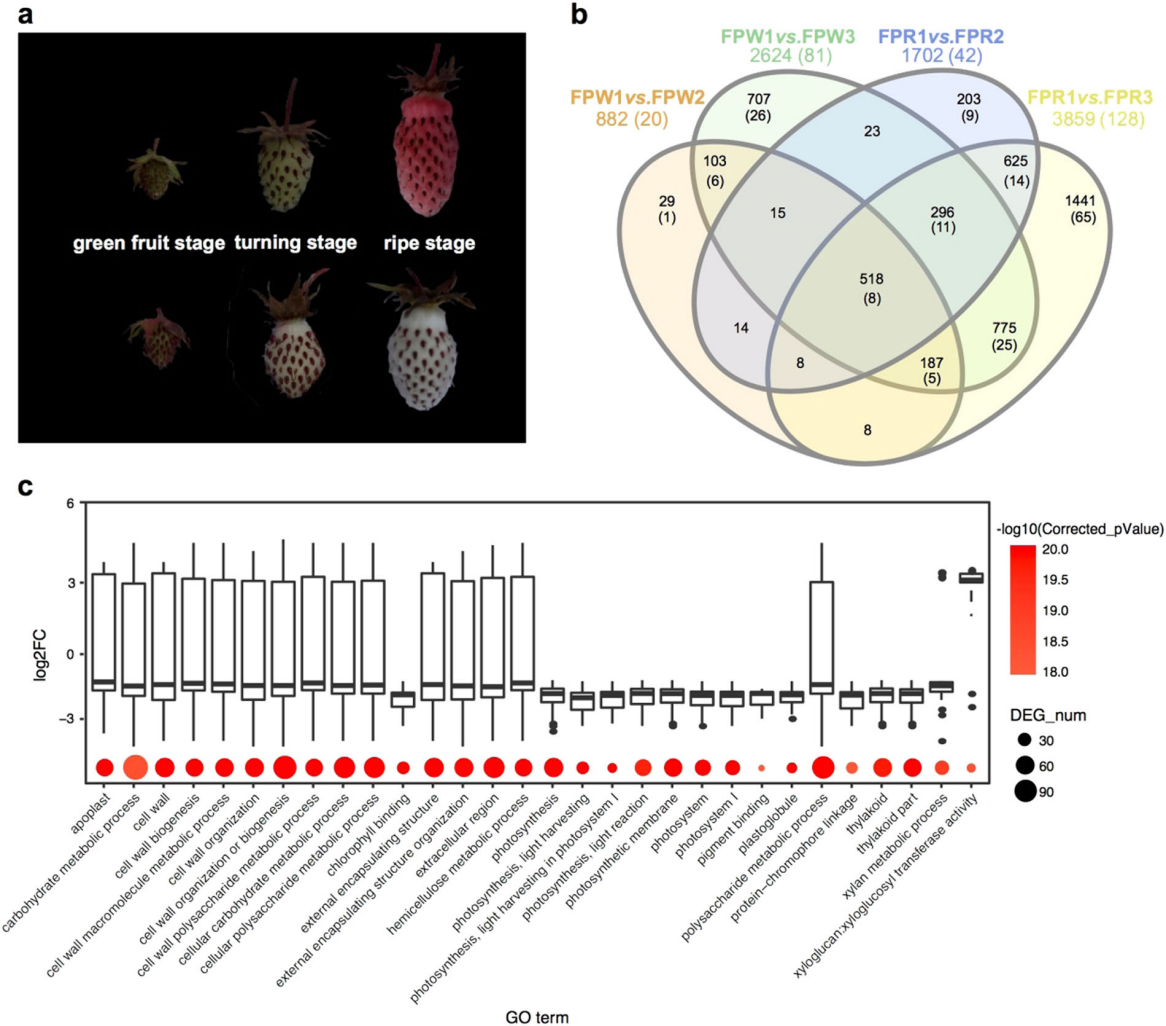
Transcriptome profiles of two typical wild varieties of *F. pentaphylla* during fruit development and ripening. **a** Color change in the fruit of two typical wild varieties of *F. pentaphylla* during fruit development and ripening. *F. pentaphylla* fruit development was divided into three stages: green fruit stage, turning stage, and ripe fruit stage. **b** Venn diagram of differentially expressed genes and lncRNAs during fruit development and ripening. The number of differentially expressed lncRNAs is indicated by bracket. **c** GO enrichment analysis of differentially expressed protein-coding genes in FPW during fruit development and ripening. The *x*-axis corresponds to GO terms, and the *y*-axis shows the log_2_ fold change of differentially expressed protein-coding genes. The color and size of the dot represent the enrichment degree and the number of differentially expressed protein-coding genes, respectively

Two typical wild varieties of *F. pentaphylla* with different fruit colors (FPR, red fruit; FPW, white fruit) were collected from the field in Sichuan Province, China. Three stages of fruit development and ripening were distinguished based on the weight and color of the fruit, which can be separated as the green fruit stage, turning stage and ripe fruit stage (Fig. 1a). Three biological replicates of each fruit sample were immediately flash-frozen in liquid nitrogen after collection in the field and stored at −80 °C for later use. Total RNAs were extracted from the collected fruit samples using an RNeasy Plant Mini Kit (Qiagen, USA) according to the manufacturer’s instructions and then subjected to RNA-seq sequencing and qRT-PCR verification. The RNA quality and quantity were determined using agarose gel electrophoresis and an Agilent 2100 Bioanalyzer (Agilent, USA). A total of 18 RNA samples (FPR and FPW at three stages, each with three biological replicates) were submitted to Chengdu Life Baseline Technology Co., Ltd. (Chengdu, China) for strand-specific library preparation and sequencing. Sequencing of the RNA-Seq libraries was performed on an Illumina HiSeq 4000 platform, producing 150 bp paired-end reads. Sequence data have been uploaded to the NCBI Short Read Archive with accession number SRP114679.

### Sequence processing, alignment, and transcript assembly

To obtain high-quality clean reads, the assessment of sequencing quality and read processing were performed by fastQC (http://www.bioinformatics.babraham.ac.uk/projects/fastqc/) and FASTX-Toolkit (http://hannonlab.cshl.edu/fastx_toolkit/). The low-quality reads were removed using the same filtering criteria as described previously^36^. The clean reads were aligned to the *F. vesca* reference genome^37^ using HISAT2 (ref. 38) with default parameters. Stringtie^38^ was used to assemble transcripts. Additionally, all unmapped reads derived from the above libraries were assembled using Trinity^39^ under default parameters to obtain comprehensive information of the *F. pentaphylla* transcriptome. Novel transcripts obtained by Stringtie and Trinity were prefixed with “SN” and “TN”, respectively. Transcriptome assembly quality was assessed by examining the RNA-Seq read representation of the assembly and searching the assembled transcripts against the database of known protein sequences. All assembled transcripts were filtered by CD-HIT^40^ (similarity > 95%) to generate a non-redundant set of transcripts. The remaining transcripts were further filtered based on expression levels. The transcripts that had an average FPKM > 1 and occurred in two or more samples were selected for further analysis. The open reading frame (ORF) of the transcript was extracted using TransDecode^39^ with default settings.

### Identification of lncRNAs and target prediction in *F. pentaphylla* during fruit development and ripening

To distinguish the lncRNAs from protein-coding transcripts, a strict filtering strategy was developed based on the criteria currently used to identify lncRNAs, comprising the following steps: (i) transcripts with lengths less than 200 bp or ORF lengths greater than 100 amino acids were excluded. (ii) Transcripts with sequence homology to existing proteins were eliminated by searching against the non-redundant protein database (NR) and the SwissProt protein database using BLASTx. (iii) The coding potential of the transcript was calculated by CPC^41^ and PLEK^42^ to remove transcripts with potential coding ability. (iv) Other classes of noncoding RNAs were removed by searching against multiple databases, including the Rfam database (http://rfam.xfam.org/), Dfam database (http://www.dfam.org/) and rRNA database (http://ssu-rrna.org/). After filtering by the above steps, the remaining transcripts were considered putative lncRNAs.

To understand the biological function of lncRNAs, the target genes were predicted based on cis-acting and trans-acting modes^42^. The sliding window strategy was used to search cis-acting target genes within 10 kb upstream and downstream of lncRNAs. To identify lncRNAs that may act in trans-acting mode, the expression correlations between lncRNAs and all protein-coding genes were calculated. LncRNAs with a trans-acting mode were recognized if Pearson’s correlation coefficient (*r*) *>* 0.9.

### Functional annotation of protein-coding genes

The protein-coding genes were compared with sequences in the NR and SwissProt protein databases to assign potential functions to genes. The programs Blast2GO^43^ and InterproScan^44^ were used to obtain GO annotations and identify protein domains, respectively. GO enrichment analysis was performed using GOseq with the following terms: biological process, molecular function, and cellular component. The metabolic pathways were predicted using KAAS^45^. KOBAS3.0 (ref. ^46^) was used for pathway enrichment analysis. Finally, genome-wide predictions of TFs in *F. pentaphylla* were performed on PlantTFDB^47^.

### Abundance estimation and differential expression analysis

The expression abundance of all genes and transcripts was estimated by RSEM^48^. Differentially expressed genes in each pairwise comparison were identified by DESeq2 (ref. 49). The absolute value of log2 ratio > 1 and the adjusted *p*-value < 0.05 were used as the thresholds to determine genes with significant expression changes. Correlation analysis among biological replicates was performed by the function corrplot in *R*. Expression patterns of differentially expressed genes were also analyzed using K-means and hierarchical clustering.

### Regulatory network construction in *F. pentaphylla* during fruit development and ripening

To gain insight into the regulatory mechanism of *F. pentaphylla* fruit development and ripening, the mRNA–lncRNA–miRNA regulatory network was constructed based on interactions and target relationships. Protein–protein interactions were analyzed by STRING^50^, and a confidence score ≥ 0.7 was set as the cut-off criterion for highly confident interactions. The miRNA sequences of *Fragaria* were widely determined in previous studies^51–53^. The target relationships of miRNA–mRNA and miRNA–lncRNA were predicted using psRNATarget^54^ with default parameters. The weighted gene co-expression network analysis (WGCNA) package was then used to build a weighted gene correlation network as described in the previous publications^55^. For the correlation analysis between the WGCNA modules and the traits, fruit colors were manually scored as discrete numerical numbers: 1, green; 2, white; 3, red, referring to Duan et al.^56^ Cytoscape^57^ was used to integrate and visualize the regulatory network.

### Validation of RNA-seq data by quantitative RT-PCR (qRT-PCR)

Ten lncRNAs and protein-coding genes were randomly selected from the present study. The cDNA synthesis, primer design (listed in Supplementary Table S1) and execution of qRT-PCR were conducted following a previous study^58^. qRT-PCR was performed on an ABI 7500 Real-Time System with three technical replicates. Statistical analysis of qRT-PCR data used the 2^−ΔΔCt^ method^59^, and the actin gene *FaACT* (GenBank accession LC017712) was used as the internal reference to normalize expression levels.

## Results and discussion

### Transcriptome profiling and genome-wide identification of lncRNA in *F. pentaphylla* fruit

Two typical wild varieties of *F. pentaphylla* with a significant difference in fruit color were found by field collection. FPR fruit showed a marked change in color from the turning stage to the ripe stage, while anthocyanins were abundant in the ripe stage. Conversely, no significant color variation in FPW fruit was observed at the late developmental stage (Fig. 1a). To investigate the underlying molecular changes during fruit development and ripening in *F. pentaphylla*, we used strand-specific sequencing to explore the expression profiles of protein-coding genes and lncRNAs for each variety at three developmental stages. After removing low-quality reads and filtering contaminant rRNA reads, a total of 186 GB high-quality clean reads were obtained from 18 samples, with 10.33 GB per sample on average (Supplementary Table S2). A relatively low rate of clean reads (61.31% on average) was mapped to the *F. vesca* reference genome, suggesting a large genomic difference between *F. pentaphylla* and *F. vesca*. Genome-guided transcriptome assembly was carried out based on uniquely mapped reads. Through the above analysis, 66,323 transcripts derived from 33,443 genes were obtained, of which 38,381 novel transcripts were detected on the basis of mapped reads. Furthermore, the unmapped reads were used to construct the full-length transcripts via de novo assembly. The two assembled transcript sets as described above were reduced to 279,129 transcripts after removing redundancy and low-expression transcripts. The remaining transcripts constituted a comprehensive transcript set and were used in subsequent analyses. The number of transcripts in *F. pentaphylla* was greater than that in the diploid strawberry model plant *F. vesca* (139,997)^60^, whereas it was close to half of the number of transcripts in *F.* *×* *ananassa* (octoploid)^61^. The relatively large difference in the number of transcripts between *F. pentaphylla* and *F. vesca* might be due mainly to genomic differences and alternative splicing. The total length of the transcripts was 207,450,409 bp, and the average length of transcripts was approximately 750 bp (Supplementary Figure S1). Among them, 56,003 transcripts had lengths longer than 1000 bp, representing 20.06% of the total transcripts (Supplementary Figure S1). The correlation analysis and dendrogram clustering illustrated the global relative relationships among the 18 samples (Supplementary Figures S2 and S3). All biological replicates clustered together with high correlation coefficients (*r* > 0.94, Supplementary Figure S2).

To predict potential lncRNAs in *F. pentaphylla* fruit, the comprehensive set of transcripts was then subjected to a specific pipeline to identify lncRNAs (see Materials and methods). As a result, 25,699 transcripts from 25,107 loci were identified as putative lncRNAs (Supplementary Figure S4a). The length of lncRNAs ranged from 201 to 2992 bp with an average length of 313 bp (Supplementary Figure S4b), which is consistent with previous analyses in plants^62–64^. In addition, lncRNAs showed lower expression levels and shorter transcript lengths than protein-coding genes (Supplementary Figure S4c). A Venn diagram revealed that only 5786 lncRNAs were expressed among all fruit samples (Supplementary Figure S4d), and a much larger proportion of lncRNAs were expressed in genotype- and stage-specific manners (Supplementary Figure S4d).

### Differential gene expression during fruit development and ripening in *F. pentaphylla*

To better understand the dynamic processes of fruit development and ripening, we evaluated the time course of development of *F. pentaphylla* fruit. We identified 882 and 2625 differentially expressed (DE) protein-coding genes in the comparisons of FPW1 *vs*. FPW2 and FPW1 *vs*. FPW3, respectively (Fig. 1b, Supplementary Table S3). Compared with the first fruit development stage, the number of DE protein-coding genes in the late stage was larger than that in the middle stage (Fig. 1b). GO enrichment analysis revealed that these DE genes in FPW were significantly enriched in important primary and secondary metabolisms, such as cell wall organization or biogenesis, photosynthesis, and response to hormone and phenylpropanoid catabolic processes (Fig. 1c). It is noteworthy that the vast majority of genes related to cell wall biogenesis and photosynthesis were downregulated in FPW2 and FPW3 compared to FPW1 (Fig. 1c). In accordance with GO enrichment analysis, the results of pathway enrichment analysis also revealed that the DE protein-coding genes were mainly involved in photosynthesis, plant hormone signal transduction and phenylpropanoid biosynthesis (Supplementary Figure S5). Furthermore, some differentially expressed genes were involved in the pigment biosynthesis pathway, including flavonoid biosynthesis (10 protein-coding genes) and carotenoid biosynthesis (13 protein-coding genes, Supplementary Figure S5). A larger number of DE genes was detected in the comparisons of FPR1 *vs*. FPR2 (1744) and FPR1 *vs*. FPR3 (3991), which reflected more intensive developmental changes in FPR (Fig. 1b). Functional annotation revealed that genes associated with photosynthesis were predominantly downregulated (Supplementary Figure S6), which was consistent with that in FPW. The development and ripening of strawberry fruit are accompanied by massive and coordinated changes in metabolic and physiological traits. Photosynthetic changes of developing strawberry fruit were different from those of the strawberry leaves, reflecting the scarcity of stomata, CO_2_ efflux, scarcity of chlorophyll, and closer chlorophyll a:b ratio^65^. Immunohistochemical and transcriptional studies revealed that the abundance of several genes involved in photosynthesis declined during fruit development in grape berry^66,67^. In this study, many protein-coding genes related to photosynthesis were repressed during fruit development and ripening (Fig. 1c). As expected, the protein-coding genes related to chlorophyll binding were also downregulated along with the fading of the green color of fruit (Fig. 1c). Numerous publications reported that changes in the cell wall occurred in strawberry during ripening^68–70^, which was consistent with what we observed in this study (Fig. 1c).

To further explore the role of lncRNAs in fruit development and ripening of *F. pentaphylla*, we investigated the lncRNA expression patterns and predicted their potential functions. As shown in Fig. 1b, more DE lncRNAs were detected in FPR when comparing FPR2 and FPR3 to FPR1, consisting of 42 and 128 DE lncRNAs. Moreover, 20 and 81 lncRNAs showed differential expression in the comparisons of FWR1 *vs*. FWR2 and FWR1 *vs*. FWR3, respectively. It is noteworthy that the number of DE lncRNAs was remarkably larger in comparison to the ripe fruit stage and green fruit stage, indicating that the late developmental stage is more complex. The function of lncRNAs in regulating fruit development and ripening has been elucidated^62,71^. Silencing of two lncRNAs in tomato resulted in an obvious delay of fruit ripening^71^. To predict the biological function of the lncRNAs, we identified lncRNA target genes and assigned them to different types of functional terms. This process identified a total of 306 DE protein-coding genes comediated by DE lncRNAs in fruit development and ripening, which were involved in important biological processes, such as photosynthesis, starch and sucrose metabolism, and diterpenoid biosynthesis (Supplementary Table S4). A considerable set of studies have demonstrated dramatic changes in the cell wall during fruit development and ripening, which directly affect fruit softening and edibility^15^. Fruit-specific rhamnogalacturonate lyase 1 (FaRGLyase1) has been confirmed to play an important role in the fruit ripening-related softening process, which is involved in the degradation of cell-wall middle lamellae and ultimately reduces strawberry firmness^72^. In the present study, *SN.315* encoding rhamnogalacturonate lyase was significantly induced at the ripe fruit stage in both of the *F. pentaphylla* varieties, whereas no lncRNA was determined to target this gene (Supplementary Table S3). Intriguingly, 47 target genes related to cell wall organization or biogenesis were co-mediated by DE lncRNAs in FPR and FPW. Of these genes, 42 (89.3%) were repressed in the late developmental stage (Supplementary Table S4). The above results suggested that lncRNAs may be involved mainly in the control of cell wall changes via repressing the expression of target genes related to cell wall organization or biogenesis. Photosynthetic pathways were also found to be repressed during fruit ripening^73^. In the present study, 32 target genes annotated as being related to photosynthesis were downregulated in the late developmental stage. Fifteen of these genes belong to the chlorophyll a-b binding protein family, and two of these genes encode a ribulose bisphosphate carboxylase small chain (Supplementary Table S4). Taken together, lncRNAs may act as negative regulators of photosynthesis and cell wall organization or biogenesis during fruit development and ripening in *F. pentaphylla*.

Increasing evidence has suggested that multiple hormones coordinate and interact with each other, forming a complex network to control early stages of fruit development and late stages of ripening. Although the entire molecular mechanisms that coordinate each hormone and the regulation of this network are largely unknown, existing investigations show that genes related to the biosynthesis and signaling of auxin and GA displayed dramatic changes through fruit development and ripening. Examples include genes related to auxin biosynthesis such as *YUCCA* (*YUC*) *flavin monooxygenases*, *tryptophan aminotransferase* (*TAA*), and GA-related genes such as *GA receptor* (*GID*), *GA 3‐oxidase* (*GA3ox*), and *GA 2-oxidase* (*GA2ox*)^5,6^. Seven *YUC* genes and five *TAA* genes exhibited different expression patterns during fruit development and ripening in both *F. pentaphylla* accessions (Supplementary Table S3), which may directly control auxin levels in *F. pentaphylla* fruits, thus permitting ripening. GID, GA3ox, and GA2ox are crucial components of the GA signaling pathway. Three transcripts encoding GID (TN118649_c0_g1, SN.8344 and SN.20031) were differentially expressed during fruit development and ripening only in the FPR accessions. In contrast, two transcripts encoding GA3ox (TN117107_c0_g1 and SN.6207) and one for GA2ox (TN131209_c0_g1) altered transcription abundance during fruit development and ripening in both *F. pentaphylla* accessions (Supplementary Table S3). Interestingly, the expression levels of *FpGA3ox* showed a progressive decline during fruit development and ripening in both accessions, whereas *FpGA2ox* was expressed at relatively high levels in the latter developmental stages (Supplementary Table S3). This finding is consistent with previous observations showing that *GA3ox* and *GA2ox* genes showed opposite expression patterns during the fruit growth and ripening period, reflecting a strategy used by the plant to fine-tune its control of bioactive GA levels^5^. Existing investigations show that the ABA/IAA ratio serves as a part of the signal to trigger fruit ripening^11^. Disruption of *9-cis-epoxycarotenoid dioxygenase* (*NCED1*), which encodes a rate-limiting enzyme involved in ABA biosynthesis, resulted in a significant decrease in ABA levels and uncolored fruits in strawberry^14^. Six genes encoding NCED1 were upregulated during fruit development and ripening in both FPW and FPR (Supplementary Table S3), which was in line with previous reports in *F. vesca*^11^. Among these genes, *SN.7771*, which shows high similarity to *FaNCED1* (JX013944.1), may be the key player involved in the magnitude of higher transcriptional activity than others and the rapid elevated response from the green fruit stage to the ripe fruit stage in FPR (Supplementary Table S3). It is noteworthy that five genes related to auxin and GA signaling pathways were targeted by lncRNAs encoding TAA (SN.16092) and GA3ox (gene06004-v1.0-hybrid, TN117107_c0_g1, SN.6206, and SN.6207), respectively (Supplementary Table S4). All these target genes were downregulated in the ripening process, while the lncRNA genes were upregulated. These data provide evidence that auxin, GA and ABA have prominent roles in fruit development and ripening in *F. pentaphylla*. Additionally, lncRNAs could alter the expression of key players related to hormone biosynthesis and be involved in the regulation of fruit development and ripening in *F. pentaphylla*.

### Identification and characterization of genes related to color changes in *F. pentaphylla* fruits

To investigate the differential regulation of key pathways between FPR and FPW, we compared gene expression patterns in FPR and FPW at three stages. In total, we identified 5621 DE genes from all comparisons (Supplementary Figure S7). Of these, 1416 protein-coding genes showed commonly differential expression in all comparisons (Supplementary Figure S7). Moreover, 2474 genes showed distinctive expression profiles in only one comparison (Supplementary Figure S7). DE genes were related to plant–pathogen interactions, phenylpropanoid biosynthesis and photosynthesis (Supplementary Figure S8). GO enrichment analysis identified 60 terms that were significantly enriched among DE genes, in which the three most enriched terms in the biological process category were “DNA integration”, “oxidation–reduction process” and “aminoglycan catabolic process” (Supplementary Figure S9). The high auxin concentration significantly inhibited the expression of anthocyanin structural genes and regulatory genes in callus cultures of red-fleshed apples, such as MYB and bHLH^9^. The auxin signaling pathway has been well studied and involves a cascade mediated through the SCF^TIR1/AFB^-Aux/IAA-ARF nuclear signaling module^74^. Among 29 DE genes involved in the auxin signaling pathway, seven genes encoding ARF, AUX/IAA, and SCF complex subunit showed distinct expression dynamics in FPR and FPW across three developmental stages (Supplementary Table S5). However, no significantly different expression was observed in these genes during the process of fruit color change, except for *SN.5270*, which was downregulated in FPR3 compared to FPR1 (Supplementary Tables S3 and S5). These results implied that the differential regulation of the auxin signaling pathway may not be the main factor contributing to the difference in fruit color in *F. pentaphylla* varieties. Additionally, transcription factors bHLH (13), MYB (12), WRKY (10) and MADS (3) were detected as significantly differentially expressed between FPR and FPW (Supplementary Table S5) and have been found to play roles in anthocyanin biosynthesis^17,18^. Of these, seven bHLH and six MYB transcription factors showed stage-specific differential expression in the ripe fruit stage, suggesting that these transcription factors may influence fruit reddening in *F. pentaphylla* (Supplementary Table S5).

The MBW complex consisting of MYB, bHLH, and WD-repeat proteins has been shown to play critical roles in regulating anthocyanin biosynthesis by controlling the expression of the anthocyanin pathway structural genes in plants^75,76^. Among them, *FaMYB10* was previously identified and shown to play critical roles in regulating the red pigment in the fruit receptacle, which was repressed by auxin and stimulated by ABA^19^. Furthermore, a specific SNP (G to C, leading to W12S variant) in *FveMYB10* was identified and then functionally confirmed to be responsible for causing the yellow fruit color in the woodland strawberry^20^. The conserved tryptophan (W) residue within the MYB protein was shown to contribute to a hydrophobic core in the DNA-binding structure^77^. Therefore, the W12S variant in the DNA-binding domain likely disrupts the DNA-binding function of MYB in *F. vesca*^20^. Transcriptomic analyses revealed that *FpMYB10* (*SN.2358*), being homologous to *FveMYB10*, was preferentially expressed in FPR (Supplementary Table S5). FpMYB10 harbors W at position 12 and exhibits a Lys (K) to Ile (I) nonsynonymous mutation at position 31 (Supplementary Figure S10). This K31I substitution at a non-conservative site was unlikely to have much of an impact on the function of MYB. A highly homologous transcript *TN118211_c0_g2* (95.54% sequence similarities to *FpMYB10*, Supplementary Figure S11) followed an expression trend similar to *FpMYB10* (Supplementary Table S3), which might also be associated with red pigment synthesis. Since a transient functional assay clearly showed that *FveMYB10* was sufficient to restore red pigments in yellow fruit accessions of *F. vesca*^20^, the relatively lower expression level of *FpMYB10* in FPW might be one contributor to the white fruit variety of *F. pentaphylla*.

**Fig. 2:**
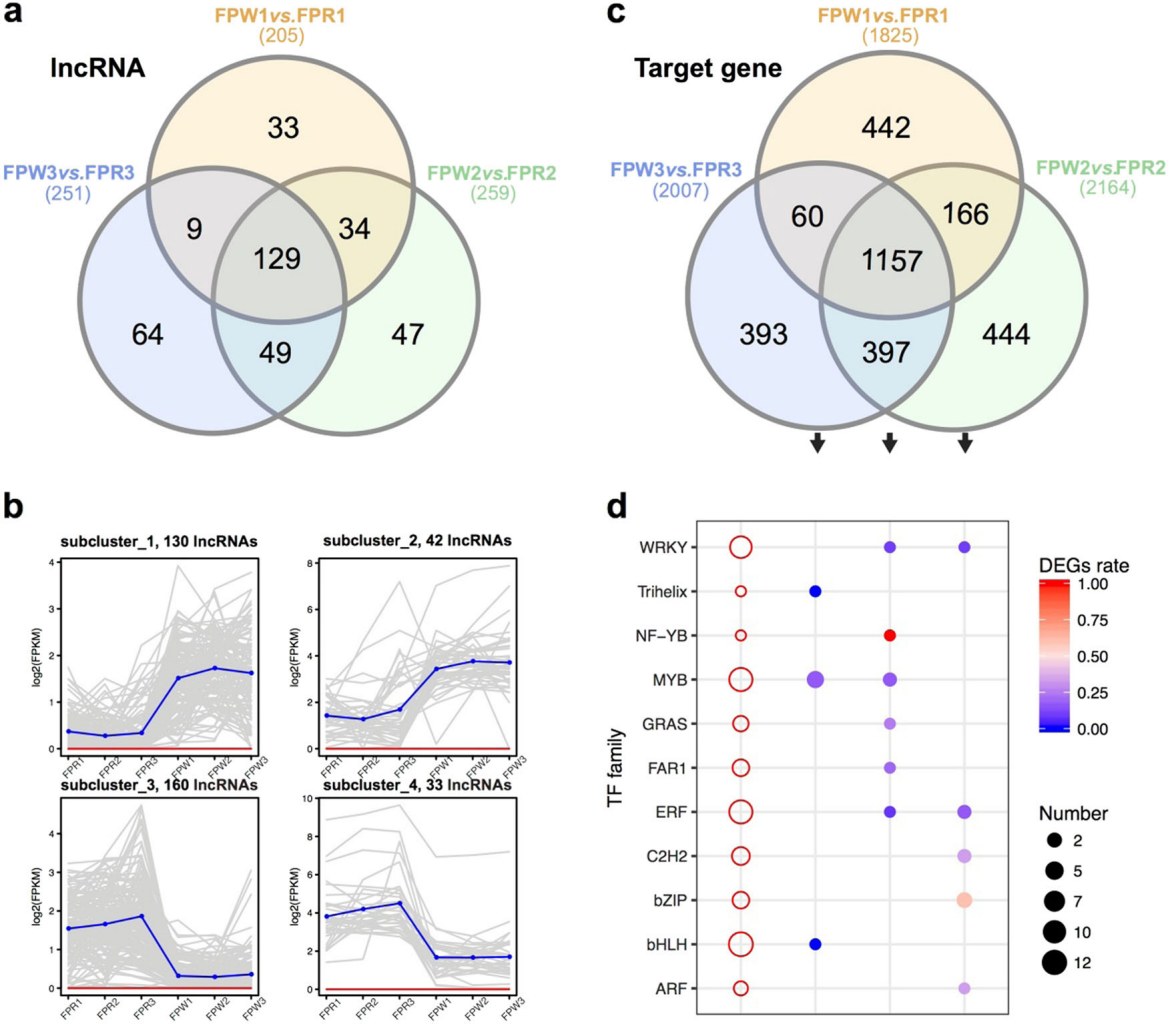
Identification and characterization of differentially expressed lncRNAs and target genes between FPW and FPR during fruit development and ripening. Three comparisons were made to quantify the differentially expressed lncRNAs and target genes at three stages. **a** Venn diagram of differentially expressed lncRNAs in FPW *vs*. FPR. **b** Expression patterns of differentially expressed lncRNAs in FPW *vs*. FPR. **c** Venn diagram of differentially expressed target genes in FPW *vs*. FPR. **d** Differential expression analysis of transcription factor (TF) families in comparisons of FPR and FPW. The x-axis represents different target gene sets, corresponding to all differentially expressed TFs (open circles) and stage-specific differentially expressed TFs (solid circles). The size of the circle represents the number of differentially expressed TFs in each TF family, and the color of the solid circle represents the rate of stage-specific differentially expressed TFs among all differentially expressed TFs

We further focused on the comparisons between FPR and FPW at three stages to characterize the specific function of lncRNAs in the color change of *F. pentaphylla* fruits. In total, 365 lncRNAs showed differential expression in at least one pairwise comparison (Fig. 2a, Supplementary Table S5). Differentially expressed lncRNAs showed stage-specific and genotype-specific expression trends across fruit development and ripening of *F. pentaphylla* (Fig. 2b). Subcluster 1 and subcluster 2, representing 172 lncRNAs, showed higher expression abundance in FPW than in FPR at all three stages (Fig. 2b). In contrast, the lncRNAs in subcluster 3 and subcluster 4 had higher expression levels in FPR than in FPW during fruit development and ripening. Previous studies have shown that lncRNAs operate not only in *cis* but also in *trans*^78,79^. According to the fundamental differences between these two categories, we used two strategies to identify DE genes that may be potential targets of these lncRNAs (see Materials and methods). In total, we identified 3059 differentially expressed target genes that were regulated by lncRNA via *cis*-acting and *trans*-acting modes (Fig. 2c). Functional annotation showed that these DE target genes were mainly associated with defense response, oxidoreductase activity, and ADP binding (Supplementary Figure S12a). They were also involved in diverse biological pathways, such as the biosynthesis of secondary metabolites, plant–pathogen interactions, and starch/sucrose metabolism (Supplementary Figure S12b).

To validate the reliability of the RNA-seq data, the expression profiles of ten randomly chosen genes were examined by qRT-PCR. As shown in Supplementary Figure S13, similar expression patterns were observed via qRT-PCR analysis as those identified in the RNA-seq data (Supplementary Figure S13).

The phenylpropanoid biosynthetic pathway genes are responsible for anthocyanin biosynthesis in plants^80^. In the present study, 30 target genes related to phenylpropanoid biosynthesis were targeted by DE lncRNAs (Table 1), encoding 4-coumarate: CoA ligase (4CL), beta-glucosidase (BGLU), cinnamyl-alcohol dehydrogenase (CAD), peroxidase (POD), caffeic acid 3-O-methyltransferase (COMT), and coniferyl-aldehyde dehydrogenase (CALDH). 4CL, as an important enzyme, plays vital roles in general phenylpropanoid metabolism and catalyzes reactions at a key branching point in the phenylpropanoid pathway^81^. Three genes encoding 4CL were potentially targeted by a single or multiple lncRNAs and showed significant expression differences between FPW and FPR, ranging from 8-fold to 32-fold, respectively (Table 1). Glycosylation is assumed to improve anthocyanin stability and provide an immense range of color variation in anthocyanins^82,83^. BGLU is also involved in anthocyanin glycosylation in addition to the well-known UDP-sugar-dependent glycosyltransferases (UGTs)^83–85^. At the ripe stage, we detected three differentially expressed *BGLU* genes, which were predicted as target genes of DE lncRNAs (Table 1). The competition of anthocyanin biosynthesis and lignin pathways for their common precursors has been demonstrated in strawberry^86^. Four genes encoding CAD and five genes encoding POD showed significant differences in expression levels between FPW and FPR (Table 1), which were also potentially targeted by DE lncRNAs. Among them, CAD is a crucial enzyme in lignin biosynthesis, and POD is associated with the metabolic interaction between anthocyanin and lignin biosynthesis^86,87^. The members of the POD family also have a function in anthocyanin degradation^88^. Based on the functional annotation, we further discovered the target genes of lncRNAs involved in plant hormone signal transduction (Supplementary Figure S14). Twenty-two of the DE lncRNA target genes widely participated in auxin, cytokinin, GA, ABA, BR, and salicylic acid (SA) signal transduction, such as ARF, cytokinin receptor (CRE) and ethylene-responsive transcription factor (ERF) (Supplementary Figure S14). In addition, many transcription factors with different expression abundances in FPW and FPR were potentially targeted by DE lncRNAs, including bHLH (13), MYB (12), ERF (12), WRKY (10), C2H2 (6) and bZIP (5) (Fig. 2d). Among them, members of the MYB, bHLH, MADS, and WRKY families were known regulatory factors of anthocyanin biosynthesis^89^. Some DE transcription factors specific to the turning stage and ripe fruit stage are presented in Fig. 2d, which may be potential targets of DE lncRNAs and involved in the control of the fading of green fruit color and the reddening of fruit, including MYB, bHLH, Trihelix, WRKY, ERF, C2H2, bZIP, and ARF (Fig. 2d). The above results suggested that the fruit development and color change in *F. pentaphylla* may be largely regulated by lncRNAs via regulation of the expression of structural genes and regulatory factors involved in phenylpropanoid and anthocyanin biosynthesis.

**Table 1:** Differentially expressed lncRNAs and target genes in phenylpropanoid pathway modulation

Gene_ID	Gene_name	Gene_description	log2 (Fold change)			lncRNA
			FPW1 *vs*. FPR1	FPW2 vs. FPR2	FPW3 vs. FPR3	
TN118390_c0_g1	*4CL*	4-coumarate:CoA ligase	-	4.78	-	TN110654_c0_g4, TN114156_c3_g2, TN118229_c2_g12, TN122782_c1_g10, TN126573_c3_g10, TN133064_c12_g2
TN123910_c0_g1	*4CL*	4-coumarate:CoA ligase	5.72	4.66	-	TN132781_c7_g1
TN125092_c0_g1	*4CL*	4-coumarate:CoA ligase	-	3.55	-	TN87879_c0_g1
gene22463-v1.0-hybrid	*BGLU*	Beta glucosidase	-	-	-	TN100144_c0_g1, TN115652_c0_g3, TN132637_c1_g3
SN.11151	*BGLU*	Beta glucosidase	-	-	−2.48	SN.7760, TN118840_c1_g18, TN123136_c3_g2, TN132483_c0_g1, TN133278_c8_g8, TN80040_c0_g1, TN96579_c0_g1
TN113907_c0_g2	*BGLU*	Beta glucosidase	-	-	−2.17	TN118840_c1_g18, TN123136_c3_g2, TN80040_c0_g1
TN117135_c3_g6	*BGLU*	Beta glucosidase	-	-	−3.08	SN.6668,SN.7760, TN118840_c1_g18, TN123136_c3_g2, TN132108_c4_g2, TN132483_c0_g1, TN80040_c0_g1
TN121899_c0_g2	*BGLU*	Beta glucosidase	-	-	-	SN.3374
TN123740_c1_g3	*BGLU*	Beta glucosidase	-	-	-	TN100144_c0_g1
SN.11440	*CAD*	Cinnamyl alcohol dehydrogenase	3.89	-	2.40	TN87879_c0_g1
SN.6919	*CAD*	Cinnamyl alcohol dehydrogenase	-	6.14	9.16	SN.3774
TN100656_c0_g1	*CAD*	Cinnamyl alcohol dehydrogenase	-	-	-	TN29352_c0_g1, TN74047_c0_g2
TN104677_c0_g1	*CAD*	Cinnamyl alcohol dehydrogenase	-	-	-	TN118229_c1_g12, TN118229_c2_g12, TN126573_c3_g10, TN128460_c0_g7, TN87879_c0_g1
TN124140_c0_g3	*CAD*	Cinnamyl alcohol dehydrogenase	-	8.24	11.09	SN.3774
TN124869_c0_g8	*CAD*	Cinnamyl alcohol dehydrogenase	-	-	−2.45	TN118518_c1_g11
TN71102_c0_g1	*CAD*	Cinnamyl-alcohol dehydrogenase	-	-	-	TN114156_c3_g2, TN118229_c1_g12, TN118229_c2_g12, TN122782_c1_g10, TN126573_c3_g10, TN128460_c0_g7, TN87879_c0_g1
TN70981_c0_g1	*CALDH*	Coniferyl-aldehyde dehydrogenase	-	-	-	TN114156_c3_g2, TN118229_c1_g12, TN118229_c2_g12, TN122782_c1_g10, TN126573_c3_g10, TN87879_c0_g1
TN119416_c0_g2	*COMT*	Caffeic acid 3-O-methyltransferase	-	-	-	TN123553_c2_g1
TN119416_c0_g6	*COMT*	Caffeic acid 3-O-methyltransferase	-	-	-	TN123553_c2_g1
SN.16569	*HCT*	Shikimate O-hydroxycinnamoyl transferase	-	-	-	SN.7984, TN133028_c1_g2
SN.1051	*POD*	Peroxidase superfamily protein	-	-	-	TN115428_c0_g1
SN.12291	*POD*	Peroxidase superfamily protein	-	−4.46	−2.46	SN.3374, TN127932_c0_g3
SN.24902	*POD*	Peroxidase superfamily protein	-	-	-	TN100144_c0_g1, TN115428_c0_g1
SN.2507	*POD*	Peroxidase superfamily protein	−2.83	−2.79	−4.32	TN133028_c1_g2
SN.3269	*POD*	Peroxidase superfamily protein	-	-	-	TN118229_c1_g12, TN128460_c0_g7
TN106277_c0_g1	*POD*	Peroxidase superfamily protein	-	-	-	TN118229_c1_g12, TN128460_c0_g7
TN112218_c0_g1	*POD*	Peroxidase superfamily protein	-	−2.18	-	TN127932_c0_g3
TN117716_c1_g1	*POD*	Peroxidase superfamily protein	-	−4.35	-	TN118518_c1_g12, TN132683_c0_g9
TN118841_c0_g1	*POD*	Peroxidase superfamily protein	−2.75	-	-	TN115428_c0_g1
TN119234_c0_g1	*POD*	Peroxidase superfamily protein	-	-	-	TN100144_c0_g1, TN115428_c0_g1

### Construction of regulatory network for fruit development and color formation

**Fig. 3:**
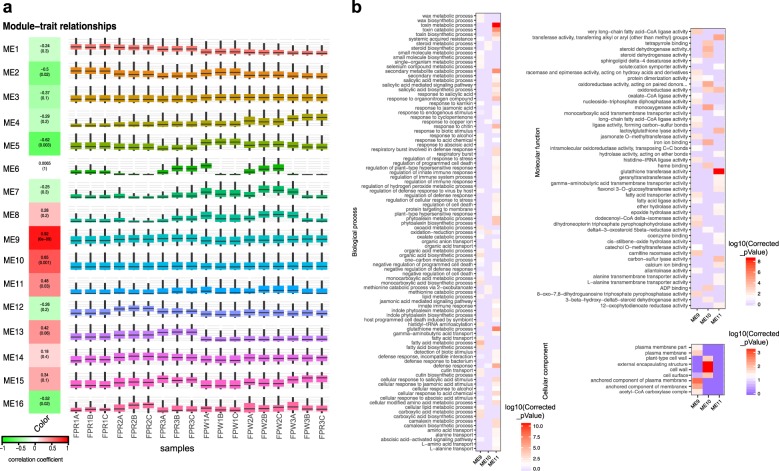
Gene co-expression module construction and functional analysis. **a** Correlations between detected modules and fruit color. The Pearson correlation coefficient is shown with the corresponding *p*-value in brackets below (left panel). The genes in different co-expression modules have distinct expression profiles (right panel). **b** Functional analysis of genes in positive correlation modules. Different colors in the block represent the different significance levels of the overrepresentation

To obtain a global view of the regulatory mechanism underlying fruit development and color changes in *F. pentaphylla*, protein-coding genes and lncRNAs were used to construct a gene co-expression network by WGCNA. In total, sixteen co-expression modules were developed, containing 15,289 elements. The Pearson correlation coefficient of each co-expression module with fruit color was calculated by WGCNA and visualized using heat maps (Fig. 3a). Out of 16 modules, module ME9 was strongly positively correlated with fruit color (*r* = 0.92). Moreover, modules ME10 and ME11 showed a relatively high correlation with fruit color (*r* = 0.65 and 0.48, respectively). To gain a further understanding of the three positive correlation modules, functional analysis of genes in these modules was conducted. The results indicated that the co-expression modules were associated with specific biological processes or cellular components. The genes in ME9 were overrepresented in the fatty acid metabolic process, carboxylic acid biosynthetic process, organic acid biosynthetic process, monocarboxylic acid biosynthetic process, oxoacid metabolic process, cutin biosynthetic process, fatty acid transport, and response to abscisic acid (Fig. 3b). For the ME10 module, key functional over-representations included the oxidation–reduction process, methionine catabolic process, and steroid metabolic process. Module ME11 was shown to be enriched in biological processes related to plant stress response, including defense response, the phytoalexin biosynthetic process, the camalexin biosynthetic process, the salicylic acid-mediated signaling pathway, and the jasmonic acid mediated signaling pathway. Differences in cell component categories further reflected functional differences between modules (Fig. 3b). In the category of cell component, the genes of ME9 were enriched in the plasma membrane and acetyl-CoA carboxylase complex (Fig. 3b). Nevertheless, the genes of the ME10 module were significantly enriched in the cell wall and cell surface (Fig. 3b).

**Fig. 4:**
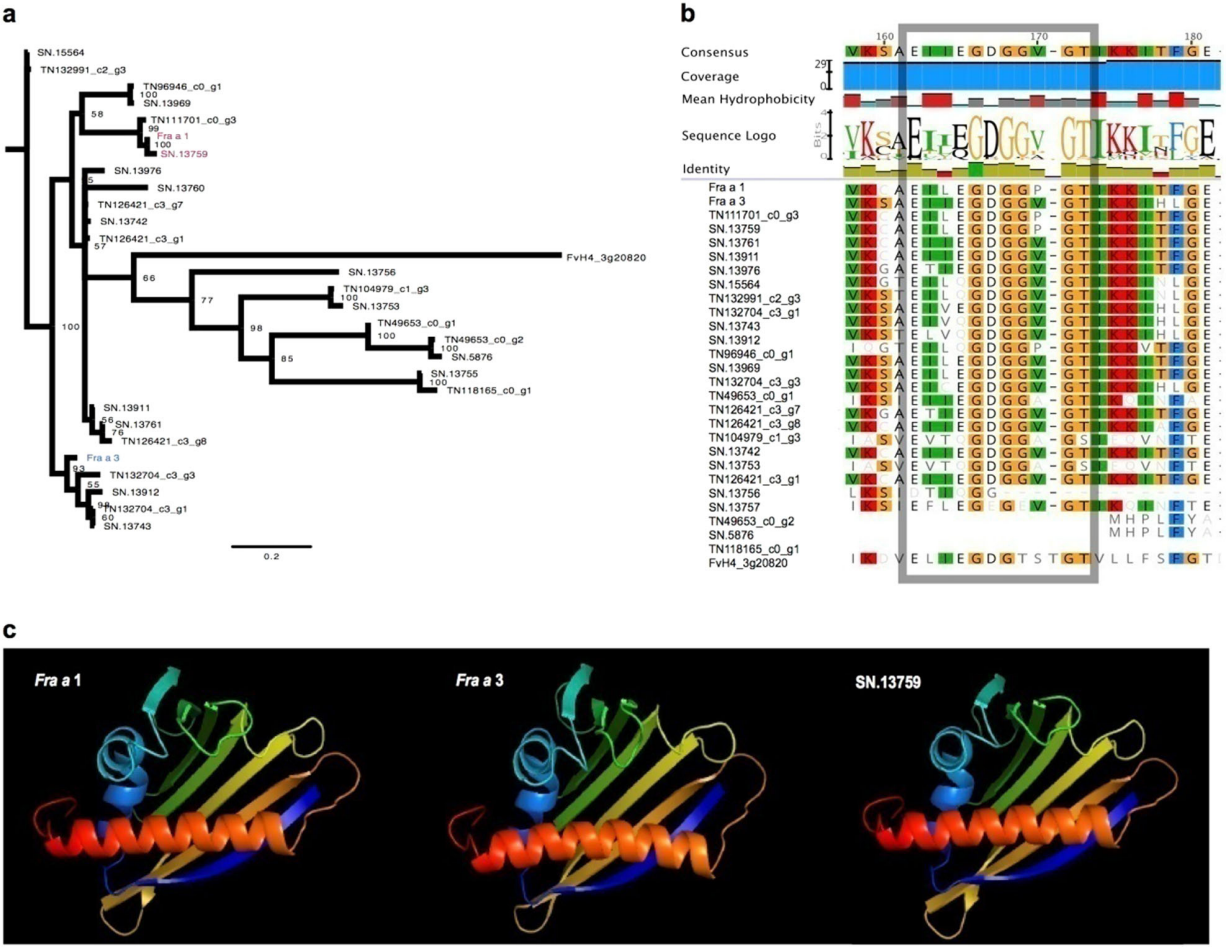
Phylogenetic analysis of the PR10 family in *F. pentaphylla*. **a** Phylogenetic analysis of PR10 family members in *F. pentaphylla*. **b** The highly conserved glycine-rich loop region of PR-10 proteins. **c** Structural features of PR-10 proteins, including *Fra a 1*, *Fra a 3*, and *SN.13759*

Close inspection revealed that genes in the co-expression module correlated with fruit color were significantly enriched in the biological processes of plant–pathogen interactions and stress responses. Multiple pathogenesis-related (PR) proteins showed distinct expression patterns between FPW and FPR, including PR10 proteins. The PR10 family is an important member of the disease-related protein family and has well-characterized functions in biotic and abiotic stress responses and plant growth and development^90,91^. Previous findings provided strong evidence that the PR10 family *Fra a* gene is directly linked to anthocyanin formation in strawberry fruit and control of color-producing flavonoid biosynthesis through binding to metabolic intermediates^21,92,93^. Silencing of *Fra a* genes led to significantly decreased levels of anthocyanins along with parallel downregulation of *phenylalanine ammonia lyase* (*PAL*) and, to a lesser extent, *chalcone synthase* (*CHS*)^22^. In this study, 29 members of the PR10 protein family were identified in *F. pentaphylla*, all of which contained the conserved Bet_v1-like domain (cd07816, Fig. 4). The members of the PR10 family contained the highly conserved glycine-rich loop sequence EG(D/N)GG(V/P)G(T/S)^91^, except for *TN49653_c0_g2, SN.5876*, and *TN118165_c0_g1* (Fig. 4). Among these identified genes, *SN.13759* was highly homologous to the *Fra a* member *Fra a 1* in *F.* *×* *ananassa* (Fig. 4). The results of cDNA amplification and SNP calling showed that the transcripts of *SN.13759* in FPR and FPW were entirely identical. *SN.13759* showed differential expression between FPW and FPR fruits, and the expression abundance was higher in FPR. The expression fold changes ranged from 1.45 to 5.12 at the three developmental stages. Notably, the expression level of *SN.13759* had a decreasing trend in FPW during fruit development and ripening, which was also observed in *F.* *×* *ananassa*^18^, whereas it decreased at first and then increased in FPR. Recently, three members of the Fra a proteins, which are essential for anthocyanin biosynthesis and red color formation in fruits, have been well studied in strawberry^22^. The transient RNAi-mediated silencing of *Fra a* resulted in significantly decreased levels of anthocyanins and upstream metabolites, which demonstrated a clear link between *Fra a* expression and anthocyanin formation^22^. *Fra a* seems to fine-tune the flavonoid composition by binding to diverse intermediates in flavonoid biosynthesis^21^. It is worth mentioning that Fra a proteins have different selectivity and affinity when binding natural metabolites in the flavonoid pathway, which might be the result of amino acid variability at certain positions^21^. Additionally, distinct expression patterns were detected in the members of the *Fra a* family in *F.* *×* *ananassa*^22^. The *Fra a 1e* and *Fra a 3* transcription levels decreased from the green fruit stage to the red fruit stage, whereas *Fra a 2* was actively transcribed in the late stages of fruit ripening^22^. Consistent with this observation, other members of the Fra a family showed different variation trends in expression levels during fruit development and ripening of *F. pentaphylla*, indicating functional differences in the members of the Fra a family.

**Fig. 5:**
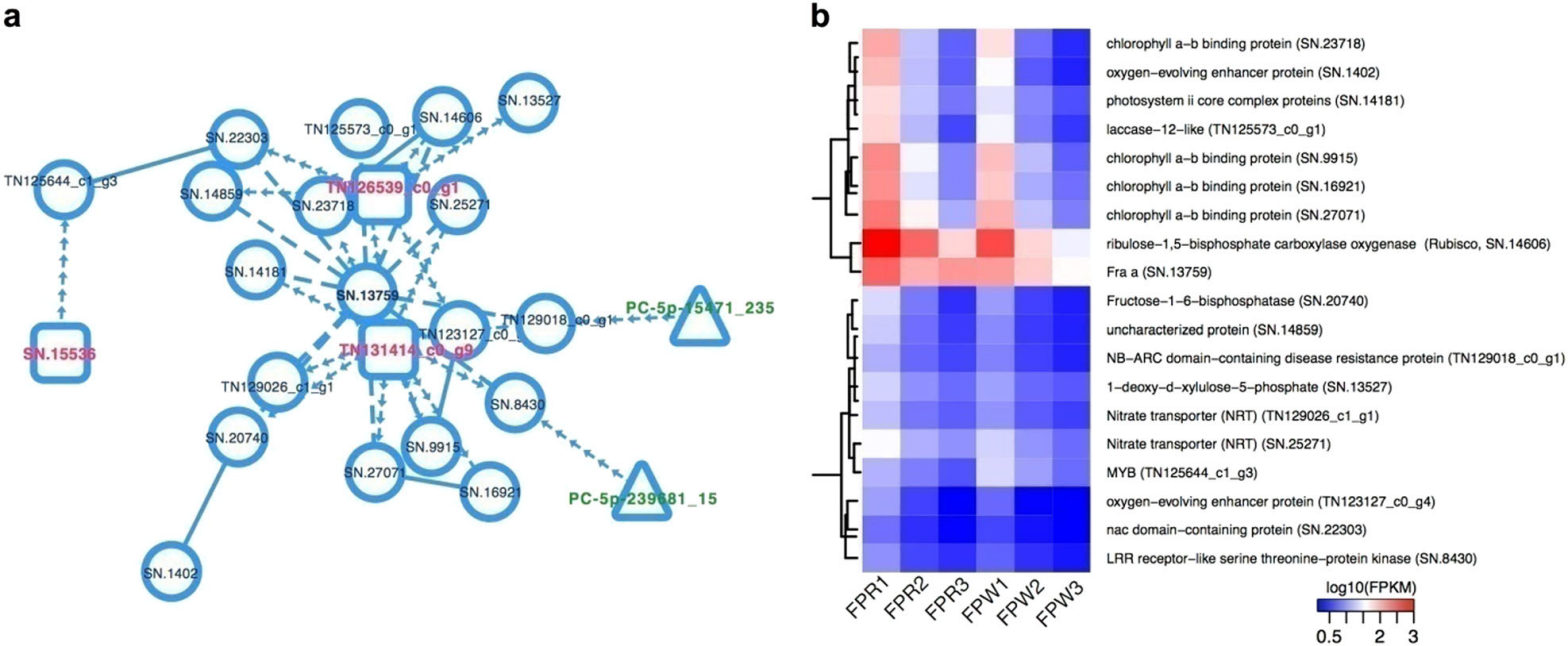
*Fra a*-dependent regulatory network in *F. pentaphylla*. **a** *Fra a*-dependent regulatory network in *F. pentaphylla*. The red, green, and black nodes denote lncRNAs, miRNAs, and protein-coding genes, respectively. The dashed arrow line denotes a target relationship between an miRNA and a protein-coding gene or lncRNA; the dashed line represents the protein–protein interaction; the solid line represents the co-expression relationship. **b** Heat map of protein-coding genes in the *Fra a*-dependent regulatory network

Despite great progress in the elucidation of *Fra a* function in anthocyanin biosynthesis, our understanding of the *Fra a*-dependent regulatory network in fruit color formation is very limited. We further intended to determine whether lncRNAs participate in this regulatory network. We attempted to construct a comprehensive regulatory network of fruit development and color formation by analyzing protein–protein interactions and target predictions of miRNA–mRNA and miRNA–lncRNA relationships. Based on the analysis above, a comprehensive regulatory network was established that contained 20,770 protein-coding genes, 3,036 lncRNAs, and 821 miRNAs. Within this network, multiple interaction patterns were recognized, such as one-to-one, one-to-many and many-to-many. We extracted a sub-network from the abovementioned regulatory network to further investigate *Fra a*-dependent regulatory relationships. A total of 24 elements were identified in this sub-network, which included nineteen protein-coding genes, three lncRNAs and two miRNAs (Fig. 5a). These protein-coding genes were associated with photosynthesis, nitrogen metabolism, and signal transduction (Fig. 5b). Coupled with fading of green and increased reddening in fruits, the expression levels of genes encoding chlorophyll a-b binding protein and ribulose-1,5-bisphosphate carboxylase oxygenase (Rubisco) were continuously downregulated in FPR and FPW fruits (Fig. 5b). Simultaneously, *SN.13759* showed a downregulatory expression trend in FPW, whereas it decreased at first and then increased in FPR (Fig. 5b). At present, it is not known whether this differential expression pattern has exactly contributed to the color variation and the mechanisms of the regulation of color variation remain unclear. Interestingly, *Fra a* expression was upregulated in naturally occurring white-fruited genotypes of *F. chiloensis* and *F. vesca*, which was likely to compensate for the low expression levels of *PAL* and *CHS* in these mutant genotypes^22^. Silencing of *Fra a* reduced *FaPAL* and *FaCHS* transcript levels by as much as 60% (ref. ^22^). In this study, the *CHS* gene *TN122753_c0_g2* was expressed at a much higher level in FPR at the ripe fruit stage than in FPW (Supplementary Table S5). It can be inferred that the deficiency of this first key enzyme leading to anthocyanin biosynthesis could contribute to colorless fruits. However, the function of the observed upregulation of *SN.13759* at the ripe fruit stage in FPR and its function related to the observed upregulation of *TN122753_c0_g2 CHS* gene might be of particular interest in future studies. Moreover, three lncRNAs (*TN126539_c0_g1*, *TN131414_c0_g9*, and *SN.15536*) targeted multiple genes in this sub-network, again indicating that lncRNAs play roles in fruit color change in *F. pentaphylla*. Moreover, we identified one uncharacterized gene that was involved in the above process (Fig. 5b). Further studies focused on the functions of these genes would greatly help to elucidate the molecular regulatory mechanisms of fruit development and color formation in *F. pentaphylla*.

## Supplementary information

Supplementary Figures S1 to S14

Supplementary Tables S1 to S5

## References

[1] Potter D (2007). Phylogeny and classification of Rosaceae. Plant Syst. Evol..

[2] Hummer KE (2007). A global conservation strategy for strawberries. Acta Hortic..

[3] Hirakawa H (2014). Dissection of the octoploid strawberry genome by deep sequencing of the genomes of *Fragaria* species. DNA Res..

[4] Liu DJ, Chen JY, Lu WJ (2011). Expression and regulation of the early auxin-responsive Aux/IAA genes during strawberry fruit development. Mol. Biol. Rep..

[5] Csukasi F (2011). Gibberellin biosynthesis and signalling during development of the strawberry receptacle. New Phytol..

[6] Kumar R, Khurana A, Sharma AK (2014). Role of plant hormones and their interplay in development and ripening of fleshy fruits. J. Exp. Bot..

[7] Symons GM (2012). Hormonal changes during non-climacteric ripening in strawberry. J. Exp. Bot..

[8] Roussos PA, Denaxa NK, Damvakaris T (2009). Strawberry fruit quality attributes after application of plant growth stimulating compounds. Sci. Hortic..

[9] Ji XH (2015). Effect of auxin, cytokinin and nitrogen on anthocyanin biosynthesis in callus cultures of red-fleshed apple (*Malus sieversii f. niedzwetzkyana*). Plant Cell Tiss. Org. Cult..

[10] Ji XH, Zhang R, Wang N, Yang L, Chen XS (2015). Transcriptome profiling reveals auxin suppressed anthocyanin biosynthesis in red-fleshed apple callus (*Malus sieversii f. niedzwetzkyana*). Plant Cell Tiss. Org. Cult..

[11] Liao X (2018). Interlinked regulatory loops of ABA catabolism and biosynthesis coordinate fruit growth and ripening in woodland strawberry. Proc. Natl Acad. Sci. USA.

[12] Kang C (2013). Genome-scale transcriptomic insights into early-stage fruit development in woodland strawberry *Fragaria vesca*. Plant Cell.

[13] Chai YM (2013). Brassinosteroid is involved in strawberry fruit ripening. Plant Growth Regul..

[14] Jia HF (2011). Abscisic acid plays an important role in the regulation of strawberry fruit ripening. Plant Physiol..

[15] McAtee P, Karim S, Schaffer R, David K (2013). A dynamic interplay between phytohormones is required for fruit development, maturation, and ripening. Front. Plant Sci..

[16] Deluc L (2006). Characterization of a grapevine R2R3-MYB transcription factor that regulates the phenylpropanoid pathway. Plant Physiol..

[17] Zhao L (2013). The R2R3-MYB, bHLH, WD40, and related transcription factors in flavonoid biosynthesis. Funct. Integr. Genom..

[18] Zhang Y (2015). Transcript quantification by RNA-Seq reveals differentially expressed genes in the red and yellow fruits of *Fragaria vesca*. PLoS. One..

[19] Medina-Puche L (2014). MYB10 plays a major role in the regulation of flavonoid/phenylpropanoid metabolism during ripening of *Fragaria* *×* *ananassa* fruits. J. Exp. Bot..

[20] Hawkins C, Caruana J, Schiksnis E, Liu Z (2016). Genome-scale DNA variant analysis and functional validation of a SNP underlying yellow fruit color in wild strawberry. Sci. Rep..

[21] Casañal A (2013). The strawberry pathogenesis-related 10 (PR-10) Fra a proteins control flavonoid biosynthesis by binding to metabolic intermediates. J. Biol. Chem..

[22] Muñoz C (2010). The strawberry fruit fra a allergen functions in flavonoid biosynthesis. Mol. Plant.

[23] Forestan C (2016). Stress-induced and epigenetic-mediated maize transcriptome regulation study by means of transcriptome reannotation and differential expression analysis. Sci. Rep..

[24] Mercer TR, Dinger ME, Mattick JS (2009). Long non-coding RNAs: Insights into functions. Nat. Rev. Genet..

[25] Liu X, Hao L, Li D, Zhu L, Hu S (2015). Long Non-coding RNAs and their biological roles in plants. Genom. Proteom. Bioinforma..

[26] Zhang YC, Chen YQ (2013). Long noncoding RNAs: New regulators in plant development. Biochem. Biophys. Res. Commun..

[27] Swiezewski S, Liu F, Magusin A, Dean C (2009). Cold-induced silencing by long antisense transcripts of an *Arabidopsis* Polycomb target. Nature.

[28] Heo JB, Sung S (2011). Vernalization-mediated epigenetic silencing by a long intronic noncoding RNA. Science.

[29] Ding J (2012). RNA-directed DNA methylation is involved in regulating photoperiod- sensitive male sterility in rice. Mol. Plant.

[30] Burleigh SH, Harrison MJ (1997). A novel gene whose expression in *Medicago truncatula* roots is suppressed in response to colonization by vesicular-arbuscular mycorrhizal (VAM) fungi and to phosphate nutrition. Plant Mol. Biol..

[31] Martín AC (2000). Influence of cytokinins on the expression of phosphate starvation responsive genes in *Arabidopsis*. Plant J..

[32] Franco-Zorrilla JM (2007). Target mimicry provides a new mechanism for regulation of microRNA activity. Nat. Genet..

[33] Shin H, Shin HS, Chen R, Harrison MJ (2006). Loss of *At4* function impacts phosphate distribution between the roots and the shoots during phosphate starvation. Plant J..

[34] Liu C, Muchhal US, Raghothama KG (1997). Differential expression of TPS11, a phosphate starvation-induced gene in tomato. Plant Mol. Biol..

[35] Wasaki J, Yonetani R, Shinano T, Kai M, Osaki M (2003). Expression of the *OsPI1* gene, cloned from rice roots using cDNA microarray, rapidly responds to phosphorus status. New Phytol..

[36] He X (2016). Comparative RNA-Seq analysis reveals that regulatory network of maize root development controls the expression of genes in response to N stress. PLoS. One..

[37] Tennessen JA, Govindarajulu R, Ashman TL, Liston A (2014). Evolutionary origins and dynamics of octoploid strawberry subgenomes revealed by dense targeted capture linkage maps. Genome Biol. Evol..

[38] Pertea M, Kim D, Pertea GM, Leek JT, Salzberg SL (2016). Transcript-level expression analysis of RNA-seq experiments with HISAT, StringTie and Ballgown. Nat. Protoc..

[39] Haas BJ (2013). De novo transcript sequence reconstruction from RNA-seq using the Trinity platform for reference generation and analysis. Nat. Protoc..

[40] Huang Y, Niu B, Gao Y, Fu L, Li W (2010). CD-HIT Suite: A web server for clustering and comparing biological sequences. Bioinformatics.

[41] Kong L (2007). CPC: Assess the protein-coding potential of transcripts using sequence features and support vector machine. Nucleic Acids Res..

[42] Li A, Zhang J, Zhou Z (2014). PLEK: A tool for predicting long non-coding RNAs and messenger RNAs based on an improved k-mer scheme. BMC Bioinforma..

[43] Conesa A (2005). Blast2GO: A universal tool for annotation, visualization and analysis in functional genomics research. Bioinformatics.

[44] Jones P (2014). InterProScan 5: Genome-scale protein function classification. Bioinformatics.

[45] Kanehisa M, Goto S, Sato Y, Furumichi M, Tanabe M (2012). KEGG for integration and interpretation of large-scale molecular data sets. Nucleic Acids Res..

[46] Xie C (2011). KOBAS 2.0: A web server for annotation and identification of enriched pathways and diseases. Nucleic Acids Res..

[47] Jin J, Zhang H, Kong L, Gao G, Luo J (2014). PlantTFDB 3.0: A portal for the functional and evolutionary study of plant transcription factors. Nucleic Acids Res..

[48] Li B, Dewey CN (2011). RSEM: accurate transcript quantification from RNA-Seq data with or without a reference genome. BMC Bioinforma..

[49] Love MI, Anders S, Huber W (2016). Differential analysis of count data - the DESeq2 package (8/8/2016). Genome Biol..

[50] Szklarczyk D (2015). STRINGv10: Protein-protein interaction networks, integrated over the tree of life. Nucleic Acids Res..

[51] Ge A (2013). Deep sequencing discovery of novel and conserved microRNAs in strawberry (*Fragaria* *×* *ananassa*). Physiol. Plant..

[52] Xu X (2013). High-throughput sequencing and degradome analysis identify miRNAs and their targets involved in fruit senescence of *Fragaria* *×* *ananassa*. PLoS. One..

[53] Li H (2013). Deep sequencing discovery of novel and conserved microRNAs in wild type and a white-flesh mutant strawberry. Planta.

[54] Dai X, Zhao PX (2011). PsRNATarget: A plant small RNA target analysis server. Nucleic Acids Res..

[55] Horvath S, Langfelder P (2008). WGCNA: an R package for weighted correlation network analysis. BMC Bioinforma..

[56] Duan NB (2017). Genome re-sequencing reveals the history of apple and supports a two-stage model for fruit enlargement. Nat. Commun..

[57] Smoot ME, Ono K, Ruscheinski J, Wang PL, Ideker T (2011). Cytoscape 2.8: New features for data integration and network visualization. Bioinformatics.

[58] Chen J, Mao L, Lu W, Ying T, Luo Z (2016). Transcriptome profiling of postharvest strawberry fruit in response to exogenous auxin and abscisic acid. Planta.

[59] KJ L, TD S (2001). Analysis of relative gene expression data using real-time quantitative PCR and the 2^(-Delta Delta C(T))^ Method. Methods.

[60] Edger PP (2018). Single-molecule sequencing and optical mapping yields an improved genome of woodland strawberry (*Fragaria vesca*) with chromosome-scale contiguity. Gigascience.

[61] Sánchez-Sevilla JF (2017). Gene expression atlas of fruit ripening and transcriptome assembly from RNA-seq data in octoploid strawberry (*Fragaria* *×* *ananassa*). Sci. Rep..

[62] Kang C, Liu Z (2015). Global identification and analysis of long non-coding RNAs in diploid strawberry *Fragaria vesca* during flower and fruit development. BMC Genom..

[63] Liu J (2012). Genome-wide analysis uncovers regulation of long intergenic noncoding RNAs in *Arabidopsis*. Plant Cell.

[64] Zhang YC (2014). Genome-wide screening and functional analysis identify a large number of long noncoding RNAs involved in the sexual reproduction of rice. Genome Biol..

[65] Blanke M (2002). Photosynthesis of strawberry fruit. Acta Hortic..

[66] Pilati S (2007). Genome-wide transcriptional analysis of grapevine berry ripening reveals a set of genes similarly modulated during three seasons and the occurrence of an oxidative burst at veraison. BMC Genom..

[67] Famiani F (2000). An immunohistochemical study of the compartmentation of metabolism during the development of grape (*Vitis vinifera* L.) berries. J. Exp. Bot..

[68] Rosli HG, Civello PM, Martínez GA (2004). Changes in cell wall composition of three *Fragaria* *×* *ananassa* cultivars with different softening rate during ripening. Plant. Physiol. Biochem..

[69] Heng Koh T, Melton LD (2002). Ripening-related changes in cell wall polysaccharides of strawberry cortical and pith tissues. Postharvest Biol. Technol..

[70] Perkins-Veazie, P. in *Horticultural Reviews* (ed. Jules Janick) Ch. 8, (John Wiley & Sons, Inc., 605 Third Avenue, New York, USA, 1995).

[71] Zhu B (2015). RNA sequencing and functional analysis implicate the regulatory role of long non-coding RNAs in tomato fruit ripening. J. Exp. Bot..

[72] Molina-Hidalgo FJ (2013). The strawberry (*Fragaria* *×* *ananassa*) fruit-specific *rhamnogalacturonate lyase 1* (*FaRGLyase1*) gene encodes an enzyme involved in the degradation of cell-wall middle lamellae. J. Exp. Bot..

[73] Wang QH (2017). Transcriptome analysis around the onset of strawberry fruit ripening uncovers an important role of oxidative phosphorylation in ripening. Sci. Rep..

[74] Wang R, Estelle M (2014). Diversity and specificity: Auxin perception and signaling through the TIR1/AFB pathway. Curr. Opin. Plant. Biol..

[75] Albert NW (2014). A Conserved network of transcriptional activators and repressors regulates anthocyanin pigmentation in eudicots. Plant Cell.

[76] Ramsay NA, Glover BJ (2005). MYB-bHLH-WD40 protein complex and the evolution of cellular diversity. Trends Plant. Sci..

[77] Ogata K (1992). Solution structure of a DNA-binding unit of Myb: a helix-turn-helix-related motif with conserved tryptophans forming a hydrophobic core. Proc. Natl. Acad. Sci. USA.

[78] Lee JT (2012). Epigenetic regulation by long noncoding RNAs. Science.

[79] Heo JB, Lee YS, Sung S (2013). Epigenetic regulation by long noncoding RNAs in plants. Chromosome Res..

[80] Zhang Y, Butelli E, Martin C (2014). Engineering anthocyanin biosynthesis in plants. Curr. Opin. Plant. Biol..

[81] Li Z, Nair SK (2015). Structural basis for specificity and flexibility in a plant 4-coumarate:CoA ligase. Structure.

[82] He J, Giusti MM (2010). Anthocyanins: Natural colorants with health-promoting properties. Annu. Rev. Food Sci. Technol..

[83] Miyahara T, Sakiyama R, Ozeki Y, Sasaki N (2013). Acyl-glucose-dependent glucosyltransferase catalyzes the final step of anthocyanin formation in. Arab. J. Plant Physiol..

[84] Schäffner AR (2016). Flavonoid biosynthesis and Arabidopsis genetics: More good music. J. Exp. Bot..

[85] Ishihara H (2016). Natural variation in flavonol accumulation in *Arabidopsis* is determined by the flavonol glucosyltransferase BGLU6. J. Exp. Bot..

[86] Ring L (2013). Metabolic interaction between anthocyanin and lignin biosynthesis is associated with peroxidase FaPRX27 in strawberry fruit. Plant Physiol..

[87] Qiao Z, A DR (2011). Transcriptional networks for lignin biosynthesis: more complex than we thought?. Trends Plant. Sci..

[88] Zhaoqi Z, Xuequn P, Zuoliang J, Yueming J (2001). Role of anthocyanin degradation in litchi pericarp browning. Food Chem..

[89] Lin-Wang K (2010). An R2R3 MYB transcription factor associated with regulation of the anthocyanin biosynthetic pathway in Rosaceae. BMC Plant Biol..

[90] Agarwal P, Agarwal PK (2014). Pathogenesis related-10 proteins are small, structurally similar but with diverse role in stress signaling. Mol. Biol. Rep..

[91] Fernandes H, Michalska K, Sikorski M, Jaskolski M (2013). Structural and functional aspects of PR-10 proteins. Febs. J..

[92] Hyun TK, Kim JS (2011). Genomic identification of putative allergen genes in woodland strawberry (*Fragaria vesca*) and mandarin orange (*Citrus clementina*). Plant OMICS.

[93] Hjernø K (2006). Down-regulation of the strawberry Bet v 1-homologous allergen in concert with the flavonoid biosynthesis pathway in colorless strawberry mutant. Proteomics.

