# Supplementary material for: Comparative transcriptome analysis uncovers the regulatory functions of long noncoding RNAs in fruit development and color changes of Fragaria pentaphylla
Source: Hortic Res. 2019 Mar 4;6:42. doi: 10.1038/s41438-019-0128-4 (PMC6397888; doi:10.1038/s41438-019-0128-4)
Supplement: Supplementary file 1 — Supplementary Figures S1 to S14 [file 41438_2019_128_MOESM1_ESM.docx]

**Supplementary Figures**

**Figure S1 Length distribution of transcripts in *F. pentaphylla.*** The histograms correspond to the number of transcripts. Total length of transcripts is represented by a solid line.

**Figure S2 Correlation analysis of 18 samples based on RNA-seq data.** The Pearson correlation coefficients for biological replicates are shown in correlation matrices.

**
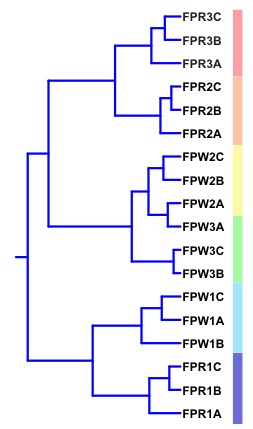
**

**Figure S3 Dendrogram clustering of 18 samples based on RNA-seq data****.** Biological replicates are marked by colored bar.

**
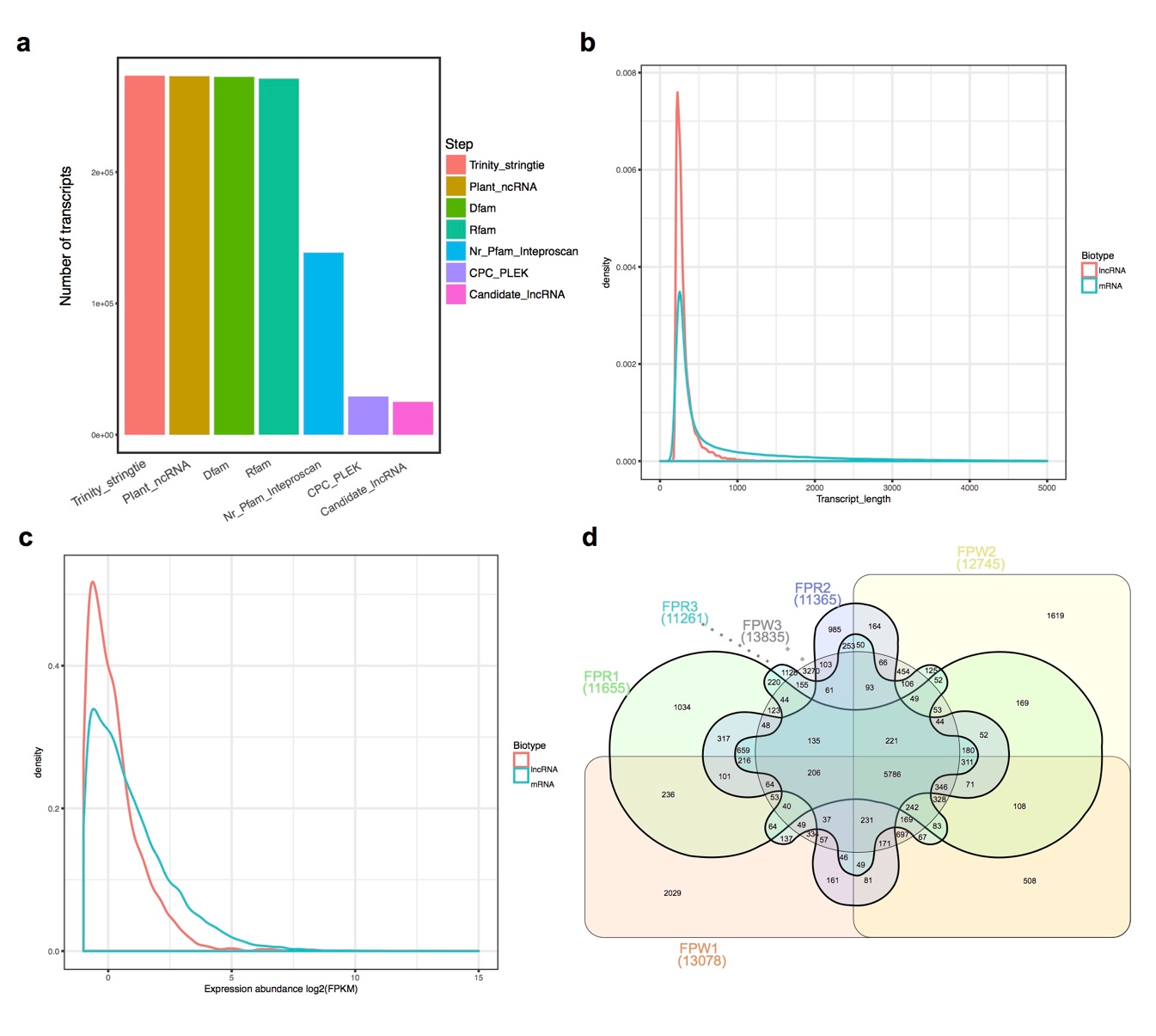
**

**Figure** **S4 Summary of lncRNAs identificationin *F. pentaphylla.* a** Four filter steps are carried out to identify lncRNAs (Details see Materials and Methods). **b** Density plot showing the range of transcript length of protein-coding genes and lncRNAs. **c** Density plot showing the range of transcript expression levels of protein-coding genes and lncRNAs. **d** Specific expression of lncRNAs in *F. pentaphylla.*

**Figure S5 KEGG pathway annotation of differentially expressed protein-coding genes in FPW during fruit development and ripening.** The y-axis corresponds to KEGG pathway, and the x-axis shows enrichment factor. The color of the dot represents the corrected p-value, and the size of the dot represents the number of differentially expressed genes mapped to the reference pathways.

**Figure S6 GO enrichment analysis of differentially expressed protein-coding genes in FPR during fruit development and ripening.** The x-axis corresponds to GO term, and the y-axis shows log_2_fold-change of differentially expressed protein-coding genes. The color and size of the dot represent enrichment degree and the number of differentially expressed protein-coding genes, respectively.


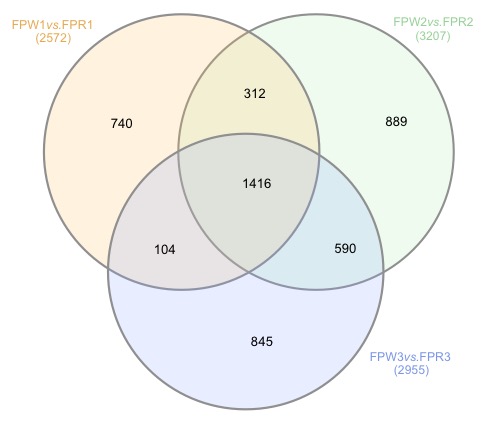


**Figure S7 Venn diagram of differentially expressed protein-coding genes between FPW and FPR during fruit development and ripening.**

**Figure S8 KEGG pathway enrichment analysis of differentially expressed** **protein-coding genes between FPW and FPR during fruit development and ripening.** The y-axis corresponds to KEGG pathway, and the x-axis shows enrichment factor. The color of the dot represents the corrected p-value, and the size of the dot represents the number of differentially expressed genes mapped to the reference pathways.

**Figure S9 GO enrichment analysis of differentially expressed protein-coding genes between** **FPW and FPR during fruit development and ripening.** Differentially expressed protein-coding genes between FPW and FPR are annotated in three GO categories: Biological process, molecular function, and cellular component. The y-axis corresponds to GO term, and the x-axis represents the number of differentially expressed protein-coding genes. Significant enrichment GO term is indicated by the asterisk.


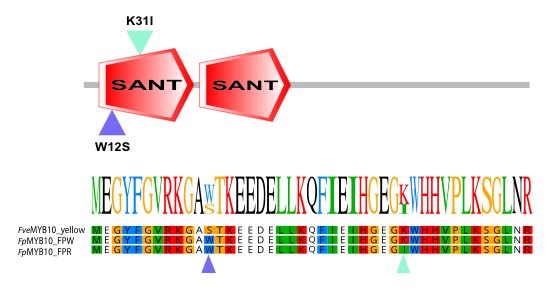


**Figure S10 Conserved domain and partial sequence alignment of candidate MYB genes.** Vertical arrow indicates the variation sites.





**Figure S11 Sequence alignment of transcripts *FpMYB10* and *TN118211_c0_g2***

**Figure S12 Functional enrichment analysis of differentially expressed target genes of lncRNAs between FPW and FPR during fruit** **development and ripening. a** GO enrichment analysis of differentially expressed target genes of lncRNAs between FPW and FPR. The y-axis corresponds to GO term, and the x-axis represents the number of differentially expressed protein-coding genes. Significant enrichment GO term is indicated by the asterisk. **b** Pathway enrichment analysis of differentially expressed target genes. The y-axis corresponds to KEGG pathway, and the x-axis shows enrichment factor. The color of the dot represents the corrected p-value, and the size of the dot represents the number of differentially expressed genes mapped to the reference pathways.


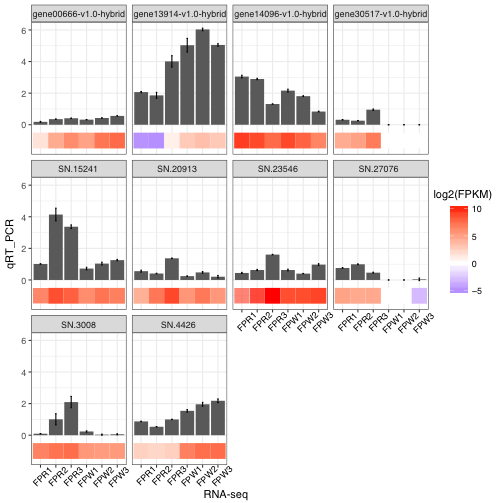


**Figure S13 qRT-PCR validation of putative lncRNAs and protein-coding genes.** The bar charts represent relative expression value measured by qRT-PCR. Color blocks under the bar charts refer to the FPKM value.


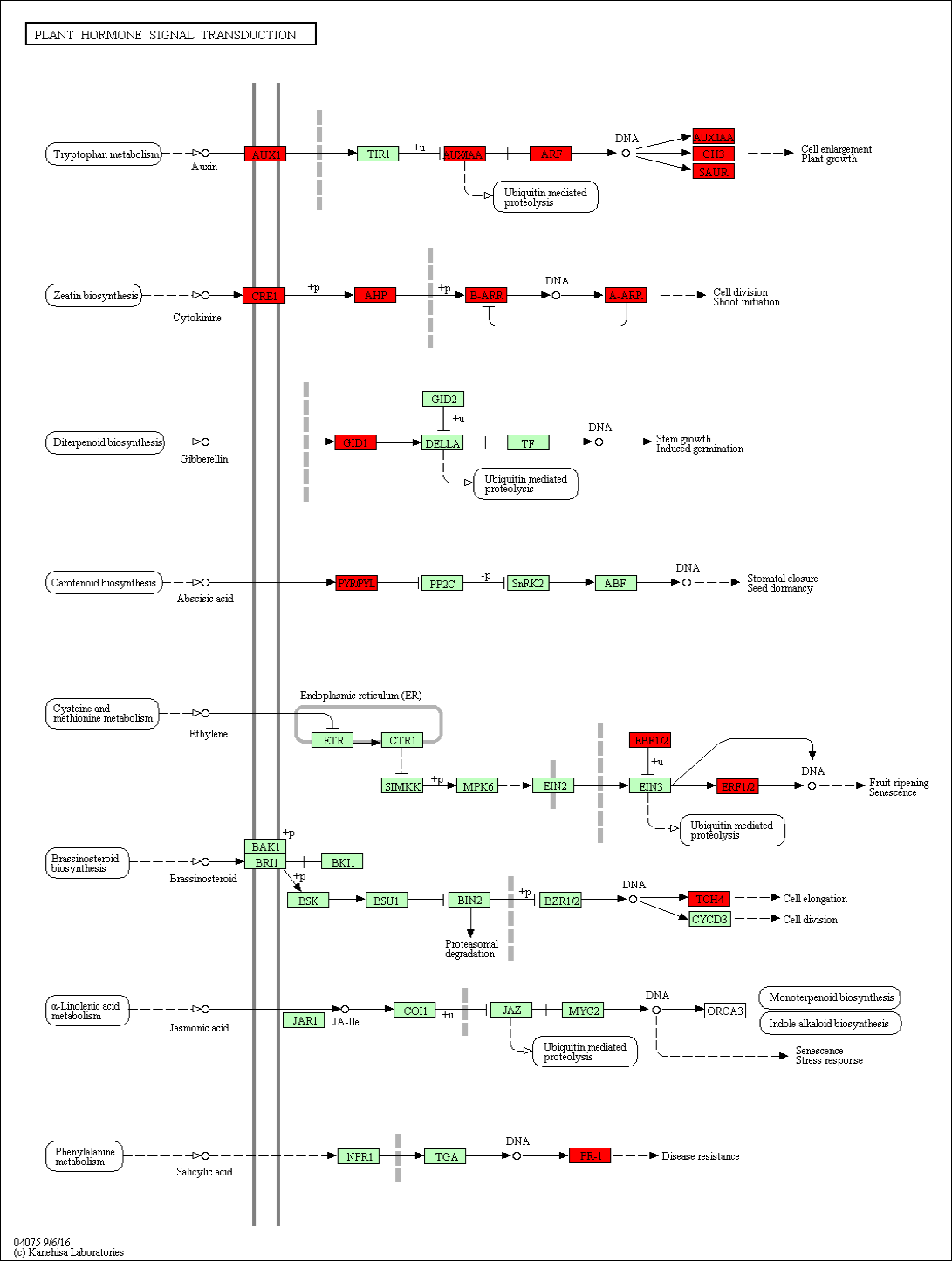


**Figure S14 Target genes of differential expression lncRNAs in plant hormone signal transduction pathway.** The red block represents target gene of differential expression lncRNAs.
